# Regulation of drug resistance and virulence of *Acinetobacter baumannii* by quorum sensing system under antibiotic pressure

**DOI:** 10.3389/fmicb.2026.1744356

**Published:** 2026-01-29

**Authors:** Xingyu Jiang, Xuchun Shan, Xiaomeng Yang, Xin Zhang, Yang Xiang, Yan Chen, Zhaohui Ni

**Affiliations:** 1 The Key Laboratory of Zoonosis, Department of Pathogen Biology, Chinese Ministry of Education, College of Basic Medical Sciences, Jilin University, Changchun, China; 2 The First Hospital of Jilin University, Changchun, China; 3 Department of Neurosurgery, The Second Hospital of Jilin University, Changchun, China

**Keywords:** *Acinetobacter baumannii*, meropenem, quorum sensing system, resistance, sub-MIC

## Abstract

**Introduction:**

*Acinetobacter baumannii* is a formidable pathogen renowned for its role in hospital-acquired infections. In recent years, largely due to antibiotic abuse and other reasons, bacteria are frequently exposed to sub-minimum inhibitory concentration (sub-MIC) levels of antibiotics. Accumulating evidence suggests that sub-MIC antibiotic pressure serves as a critical driver of bacterial resistance evolution and virulence adaptation. However, the regulatory mechanisms underlying antibiotic stress adaptation in *A. baumannii* remains poorly understood. The quorum sensing (QS) system is a key bacterial signaling network that senses population density and coordinates vital physiological functions and environmental adaptations. Targeting QS system to attenuate virulence and resistance represents a promising strategy for combating multi-drug-resistant infections. Nevertheless, the role of systems in regulating antibiotic stress response in *A. baumannii* has not been elucidated.

**Methods:**

In this study, we used the wild-type (WT) strain of *A. baumannii* and an isogenic *abaI* deletion mutant strain (Δ*abaI*) to investigate the involvement of QS in adaptive responses under meropenem sub-MIC pressure. The analysis was performed by phenotypic experiments such as bacterial biofilm formation and motility detection, transcriptome sequencing (RNA-seq) and qRT-PCR verification.

**Results:**

We found that under antibiotic pressure, the WT strain developed significantly enhanced resistance, accompanied by increased biofilm formation, surface motility, adherence to and invasion of A549 cells, and pathogenicity in *Galleria mellonella*. In contrast, the Δ*abaI* strain showed no significant changes in resistance, motility, host cell adhesion and invasion, or virulence, with all these parameters remaining substantially lower than those of the antibiotic-treated WT. Interestingly, biofilm formation was still significantly enhanced in the Δ*abaI* strain, suggesting compensatory activation of alternative regulatory mechanisms. Transcriptomic analysis revealed that sub-MIC meropenem triggered extensive gene expression changes in both the WT and Δ*abaI* strains. In the WT, differentially expressed genes were enriched in pathways including quorum sensing, biofilm formation, ABC transporters, and two-component systems. In contrast, the Δ*abaI* mutant exhibited distinct transcriptional profiles, with enrichment in Δ-lactam resistance, aromatic amino acid biosynthesis, and metabolite transport. The expression trends of key virulence- and resistance-associated genes were further validated by qRT-PCR, confirming the reliability of the RNA-seq data.

**Discussion:**

Our study underscores the potential of targeting the QS system to mitigate antibiotic-driven adaptation and provides a strategic basis for controlling multidrug-resistant *A. baumannii* infections.

## 1 Introduction

*Acinetobacter baumannii* is a formidable Gram-negative pathogen notorious for causing severe hospital-acquired infections, and it is associated with a broad spectrum of diseases, including ventilator-associated pneumonia (VAP), bacteremia, skin and soft tissue infections (McConnell et al., 2013; Scribano et al., 2024). The species exhibits a remarkable capacity for resistance evolution, often leading to the emergence of multidrug-resistant (MDR) strains. Among these, carbapenem-resistant *A. baumannii* (CRAB) represents a particularly alarming threat and has been designated by the World Health Organization (WHO) as a critical-priority pathogen necessitating urgent therapeutic solutions (Joly-Guillou, 2005; Antunes et al., 2014).

Carbapenems, tetracyclines, quinolones, and aminoglycosides are among the antibiotics commonly used in the treatment of *A. baumannii* infections. However, the extensive and frequently indiscriminate use of these agents often leads to the accumulation of sub-inhibitory concentrations (sub-MIC) of antibiotics in both clinical and environmental settings. Exposure to sub-MIC levels can trigger profound bacterial stress responses, ultimately accelerating the evolution of resistance (Dal Sasso et al., 2003; Yang et al., 2020). Certain sub-MIC exposures have been shown to increase bacterial mutation rates and activate efflux pumps, further promoting resistance mechanisms (Gutierrez et al., 2013; Cortes et al., 2008). Additionally, sub-MIC antibiotics may modulate the expression of virulence factors, thereby enhancing host colonization and immune evasion (Chen et al., 2021; Viswanathan, 2014). Despite these observations, the molecular mechanisms governing *A. baumannii* adaptation under sub-MIC stress remain poorly understood.

Quorum sensing (QS) represents a key cell–cell communication system that enables bacteria to coordinate gene expression in response to population density, thereby synchronizing collective behaviors such as virulence factor production, biofilm formation, and antibiotic resistance (Papenfort and Bassler, 2016). In *A. baumannii*, this regulatory network is primarily governed by the AbaI/AbaR two-component system, which plays a pivotal role in modulating pathogenicity and antimicrobial resistance (Oh and Han, 2020). It has been demonstrated that the AbaI/AbaR system regulates biofilm formation, surface motility, and significantly contributes to antibiotic tolerance (Dou et al., 2017). Moreover, QS in *A. baumannii* engages in cross-talk with other regulatory systems, such as BfmRS, forming an integrated signaling circuit that enhances biofilm architecture and stress resilience (Sun et al., 2021). This interconnection suggests that QS acts as a central modulator within a broader regulatory network, fine-tuning bacterial adaptation in response to environmental challenges.

Accumulating evidence indicates that sub-MIC antibiotic exposure serves as a major driver of resistance development and virulence evolution in bacteria, profoundly affecting treatment efficacy and facilitating persistent, difficult-to-eradicate infections (Andersson and Hughes, 2014). Nevertheless, the regulatory mechanisms behind these adaptations—particularly the role of QS—in *A. baumannii* remain inadequately defined. A critical unresolved question is how the AbaI/AbaR system perceives and integrates environmental antibiotic signals to globally modulate bacterial adaptability, virulence gene expression, and resistance evolution.

To address this knowledge gap, we investigated the role of the AbaI/AbaR QS system in mediating adaptive responses of *A. baumannii* to sub-MIC meropenem exposure. Using the wild-type ATCC 17978 and its isogenic Δ*abaI* mutant, we performed comparative phenotypic assays and transcriptomic analyses to identify key genetic pathways modulated by QS under antibiotic stress. Our study provides mechanistic insights into how *A. baumannii* thrives under antibiotic pressure and how QS orchestrates global adaptive changes affecting virulence and resistance evolution, thereby offering new perspectives for combating infections caused by this resilient pathogen.

## 2 Materials and methods

### 2.1 Bacterial strains

*A. baumannii* wild-type strain ATCC 17978, an *abaI* mutant strain (Δ*abaI*) and *abaI* complemented strain (Δ*abaI* (pME*abaI*)) were used in this study. The wild strain was kindly provided by Dr. Ayush Kumar (University of Manitoba, Canada), the Δ*abaI* mutant and Δ*abaI* (pME*abaI*) mutant was generated in our laboratory using established methods (Sun et al., 2021). Bacterial strains used in this study are listed in Supplementary Table S1. For drug susceptibility testing, *Escherichia coli* ATCC 25922 obtained from the Department of Pathogen Biology at Jilin University, China, was used as a reference strain.

### 2.2 Bacterial culture conditions

All strains were inoculated in Luria-Bertani (LB) broth (Gibco, United States) and cultured at 37 °C with shaking at 200 rpm under dark conditions until logarithmic growth phase was reached. Cultures from this phase were used for subsequent experiments. For the sub-MICs treatment groups, WT and ∆*abaI* were transferred to 5 mL of LB broth containing meropenem at a concentration equivalent to 1/2 MIC. In the ∆*abaI* sub-MIC+AHL group, the N-3-hydroxy-dodecanoyl-homoserine lactone (3-OH-C12-HSL; Sigma, United States) was dissolved in DMSO and supplemented into the to the meropenem-containing LB broth at a concentration of 10 μM. As a control, pure DMSO without AHL was added to a medium volume of control cultures.

### 2.3 MIC measurement

The minimum inhibitory concentration (MIC) of meropenem was determined for both WT and ∆*abaI* strains using the broth microdilution method in 96-well plates. Bacterial suspensions were adjusted to approximately 1 × 10^7^ colony forming units (CFUs)/mL in LB broth and added to wells containing serial twofold dilutions of meropenem, with final concentrations ranging from 0 to 32 μg/mL (0, 0.0625, 0.125, 0.25, 0.5, 1, 2, 4, 8, 16, 32 μg/mL). The plates were incubated at 37 °C for 24 h. Bacterial growth was monitored by measuring the optical density at 590 nm (OD_590_) of the MIC was defined as the lowest concentration of meropenem that completely inhibited visible growth, in accordance with the Clinical and Laboratory Standards Institue (CLSI) 2022 guidelines. All experiments were performed in at least three independent replicates.

### 2.4 Growth curve measurement

Growth curves were determined for both the WT and ∆*abaI* strains using a 96-well microplate assay. Aliquots (1 mL) were taken from overnight cultures of each strain, and 20 μL of 0.5 McFarland standard bacterial suspension was transferred into wells containing 180 μL of LB broth. For the sub-MIC treatment groups, meropenem was added to a final concentration of 1/2 MIC. The plate was incubated at 37 °C, and the OD_590_ of cultures was measured at hourly intervals over a 36-hourperiod to draw the growth curve. All growth experiments were performed with three independent biological replicates.

### 2.5 Drug resistance evolution experiment

To investigate the evolution of antibiotic resistance, WT and ∆*abaI* strains were serially passaged under meropenem pressure. Aliquots (20 μL) of each bacterial culture were inoculated into 180 μL LB broth containing meropenem at 1/2 MIC (0.5 μg/mL for WT and 0.25 μg/mL for ∆*abaI*), resulting in an initial bacterial concentration of approximately 1 × 10^7^ CFU/mL. Cultures were incubated at 37 °C and 200 rpm for 24 h per cycle. After each cycle, 20 μL of culture was transferred into fresh LB liquid medium containing the corresponding concentration of meropenem for subculture. This passaging was repeated for seven cycles to establish the antibiotic pressure groups. Parallel control groups for both strains were passages in meropenem-free LB medium for the same number of cycles. The MIC of meropenem was determined for each strain at the end of every passage cycle. All experiments were performed in three independent biological replicates.

### 2.6 Surface motility assay

The surface motility of each strain was assessed using a previously described method with minor modifications (Corral et al., 2021). Overnight cultures of each strain were adjusted to a concentration of 1 × 10^7^ CFU/mL in LB broth. For the sub-MIC treatment groups, WT, Δ*abaI* and ∆*abaI* +AHL strains were cultured overnight in LB broth containing 1/2 MIC meropenem as described in Section 2.2. A 10 μL aliquot of each bacterial suspension was spot-inoculated at the center of motility assay plates composed of 10 g/L tryptone, 5 g/L NaCl, and 0.3% Noble agar. The plates were air-dried at room temperature for 1–2 h and then incubated at 37 °C for 12 h. The diameter of bacterial surface expansion was measured to quantify motility.

### 2.7 Antibiotic-induced biofilm formation assays

Biofilm formation under antibiotic exposure was quantified using 96-well microtiter plates according to a previously described method (He et al., 2015) with minor modifications. Bacterial strains were inoculated at approximately 1 × 10^6^ CFU/mL in LB broth containing meropenem at sub-MIC (1/2 MIC) or without antibiotic, followed by incubation at 37 °C for 24 h. After incubation, the OD of the planktonic culture was measured at 590 nm (OD_Planktonic_). The adhered biofilm was then stained with 1% crystal violet for 30 min, washed three times with distilled water, air-dried for 1 h, and solubilized with 95% ethanol. The OD of the dissolved crystal violet was measured at 590 nm (OD_CV Biofilm_). The biofilm formation index was calculated as: [BFI = (OD_CVBiofilm_-OD_CV Control_)/OD_Planktonic_]. The relative biofilm formation index (RBFI) was determined using the wild-type (WT) strain without antibiotic treatment as the positive reference: [RBFI = (BFI_treatment_/BFI_WT_)].

### 2.8 Cell culture and infection

The human alveolar epithelial cell line A549 was obtained from the Department of Pathogen Biology (Jilin University, China). Cells were routinely cultured in Dulbecco’s Modified Eagle Medium (DMEM; Gibco, China) supplemented with 10% heat-inactivated fetal bovine serum (FBS; Gibco, Australia), 50 U/mL penicillin, and 50 μg/mL streptomycin (both from Gibco, China). For infection assays, A549 cells were grown to 90% confluency, after which were added at a multiplicity of infection (MOI) of 10 in fresh DMEM (without antibiotics) and incubated at 37 ° C under 5% CO_2_.

### 2.9 A549 adhesion and invasion assays

A549 Cells were seeded into 6-well plates and cultured until 90% confluency was reached. *A. baumannii* strain were grown overnight and adjusted to MOI of 10. Cells were infected in DMEM without FBS or antibiotics and incubated at 37 °C under 5% CO_2_ for 2 h. For the adhesion assay, infected monolayers were washed three times with PBS and then lysed with 500 μL of 0.1% Triton X-100. For the invasion assay, extracellular bacteria were eliminated by treating the infected cells with 500 μg/mL gentamicin for 30 min, followed by cell lysis using 500 μL of 0.1% Triton X-100 to release intracellular bacteria. In both assays, the lysates were serially diluted, plated on LB agar, and incubated at 37 °C for 24 h before enumerating bacterial colony-forming units (CFUs).

### 2.10 *Galleria mellonella* infection and killing assays

The virulence of *A. baumannii* strains was evaluated using the *Galleria mellonella* as an in-infection model. Bacteria were cultured to the exponential growth phase, washed two to three times with phosphate-buffered saline (PBS), and adjusted to a turbidity equivalent to the 0.5 McFarland standard using PBS. For each bacterial strain, 16 larvae of uniform size were randomly selected. A 20 μL bacterial suspension (approximately 3 × 10^6^ CFU/larva) was injected into the left or right hind leg of each larvae using a sterile syringe. A control group of 16 larvae was injected with 20 μL of PBS. After injection, larvae were incubated in the dark at 37 °C for 5 days. Mortality was recorded daily, and a larva was considered dead if it displayed no movement in response to gentle tactile stimulation.

### 2.11 Mouse infection experiment

The mouse infection experiments strictly followed the ethical guidelines issued by the Animal Protection and Utilization Committee of Jilin University (Protocol Number: 2025-637) and was approved by the Animal Experiment Ethics Committee of College of Basic Medical Sciences, Jilin University. Six-week-old female BALB/C mice were used in this study. All mice were raised in the Animal Biosafety Secondary Laboratory of Jilin University (ABSL-2). *A. baumanni*-mouse infection model was established according to the published methods (Harris et al., 2013). Specifically, approximately 1 × 10^7^ colony-forming units (CFU) of bacteria were suspended in 100 μL of saline and infected by intraperitoneal injection.

### 2.12 RNA preparation and qRT-PCR

Total RNA was extracted from *A. baumannii* cultures using the RNAprep Pure Culture Bacterial Total RNA Extraction Kit (Tiangen, Beijing, China), according to the manufacturer’s instructions. Reverse transcription was performed using FastKing one-step genomic first strand Removal cDNA synthesis kit (Tiangen, Beijing, China). Quantitative real-time PCR (qRT-PCR) was carried out with Taq Pro Universal SYBR qPCR Master Mix (Vazyme, Nanjing, China) on an ABI 7300 Plus Real-Time PCR system (Applied Biosystems, United States). All primer sequences used are listed in Supplementary Table S2. Gene expression levels were calculated using the 2^−ΔΔCt^ method.

### 2.13 RNA sequencing and transcriptome analysis

Total RNA was extracted from bacterial cultures using Trizol Reagent (Invitrogen, Shanghai, China). RNA quality and integrity were assessed with a NanoDrop spectrophotometer (Thermo Scientific, United States) and a Bioanalyzer 2100 system (Agilent, United States). Ribosomal RNA was removed from total RNA using the Zymo-Seq RiboFree Total RNA Library Kit. First-strand cDNA was synthesized with random hexamer primers and SuperScript III reverse transcriptase, followed by second-strand synthesis using DNA Polymerase I and RNase H. cDNA fragments of 400–500 bp were selected and purified with the AMPure XP system (Beckman Coulter, Beverly, CA, United States) and quantified using the Agilent high sensitivity DNA kit on the Bioanalyzer 2100. Sequencing libraries were constructed and paired-end sequenced on an Illumina NovaSeq 6000 platform by Shanghai Personal Biotechnology (Shanghai, China).

### 2.14 Statistical analysis

All statistical analyses were performed using SPSS software (version 24.0). Data are presented as mean ± standard error of the mean (SEM). Differences between two groups were assessed using the student’s *t*-test, while comparisons among multiple groups were conducted by one-way analysis of variance (ANOVA). A *P*-value < 0.05 was considered statistically significant for all tests.

## 3 Results

### 3.1 QS regulates the evolution and growth rate of *Acinetobacter baumannii* under antibiotic pressure

**Figure 1:**
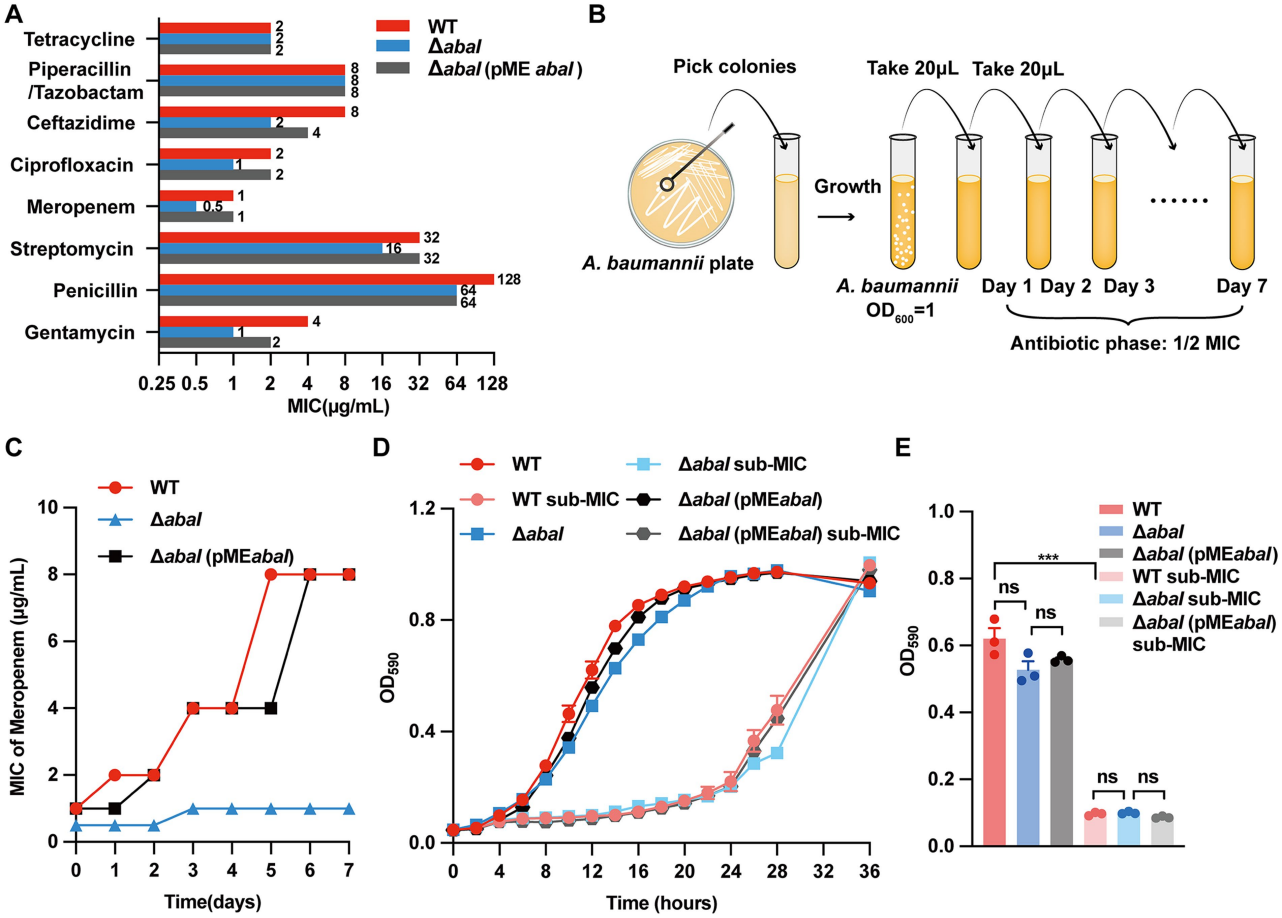
QS regulates the evolution and growth rate of *A. baumannii* under antibiotic pressure. **(A)** MICs of 8 antibiotics against WT, Δ*abaI*, and Δ*abaI*(pME*abaI*); **(B)** Flow chart of resistance evolution in *A. baumannii*; **(C)** Effects of QS on the evolution of bacterial resistance under antibiotic pressure; **(D)** Growth curve of bacteria; **(E)** OD value of bacterial culture medium at 12 h. The data are presented as the mean ± SEM. ***p < 0.001, ns: non-significant.

The minimum inhibitory concentrations (MICs) of different antimicrobials were first determined for the wild-type (WT), the Δ*abaI* mutant and *abaI* complemented strain (Δ*abaI*(pME*abaI*)). Deletion of the QS synthase gene *abaI* significantly lowered the MICs of gentamicin, penicillin, streptomycin, meropenem, ciprofloxacin and ceftazidime (Figure 1A; Supplementary Table S3), however, the Δ*abaI*(pME*abaI*) complemented strain was similar to the WT strain, confirming that quorum sensing contributes to the intrinsic antibiotic susceptibility in *A. baumannii*. Because carbapenems are the first-line agents against this pathogen, a sub-inhibitory concentration of meropenem was subsequently applied as selective pressure in a 7-day evolution experiment (Figure 1B). During the experiment, the meropenem MIC for the WT strain rose from 1 to 8 μg/mL, whereas the MIC for the *ΔabaI* mutant increased only from 0.5 to 1 μg/mL, and the MIC for the Δ*abaI*(pME*abaI*) complemented strain rose from 1 to 8 μg/mL, was similar to the WT strain (Figure 1C). Consequently, the WT population evolved from sensitivity to high-level resistance, whereas the QS-deficient population remained susceptible, indicating that the absence of quorum sensing markedly attenuates resistance evolution.

Growth kinetics revealed no significant difference between WT, Δ*abaI* and Δ*abaI*(pME*abaI*) under antibiotic-free conditions. When meropenem was present, the strains exhibited a comparable reduction in growth rate (Figures 1D,E). These data indicate that quorum-sensing inhibition does not compromise bacterial viability, enabling modulation of resistance or virulence without imposing selective pressure for resistance development.

### 3.2 Effects of QS on bacterial surface motility under antibiotic pressure

**Figure 2:**
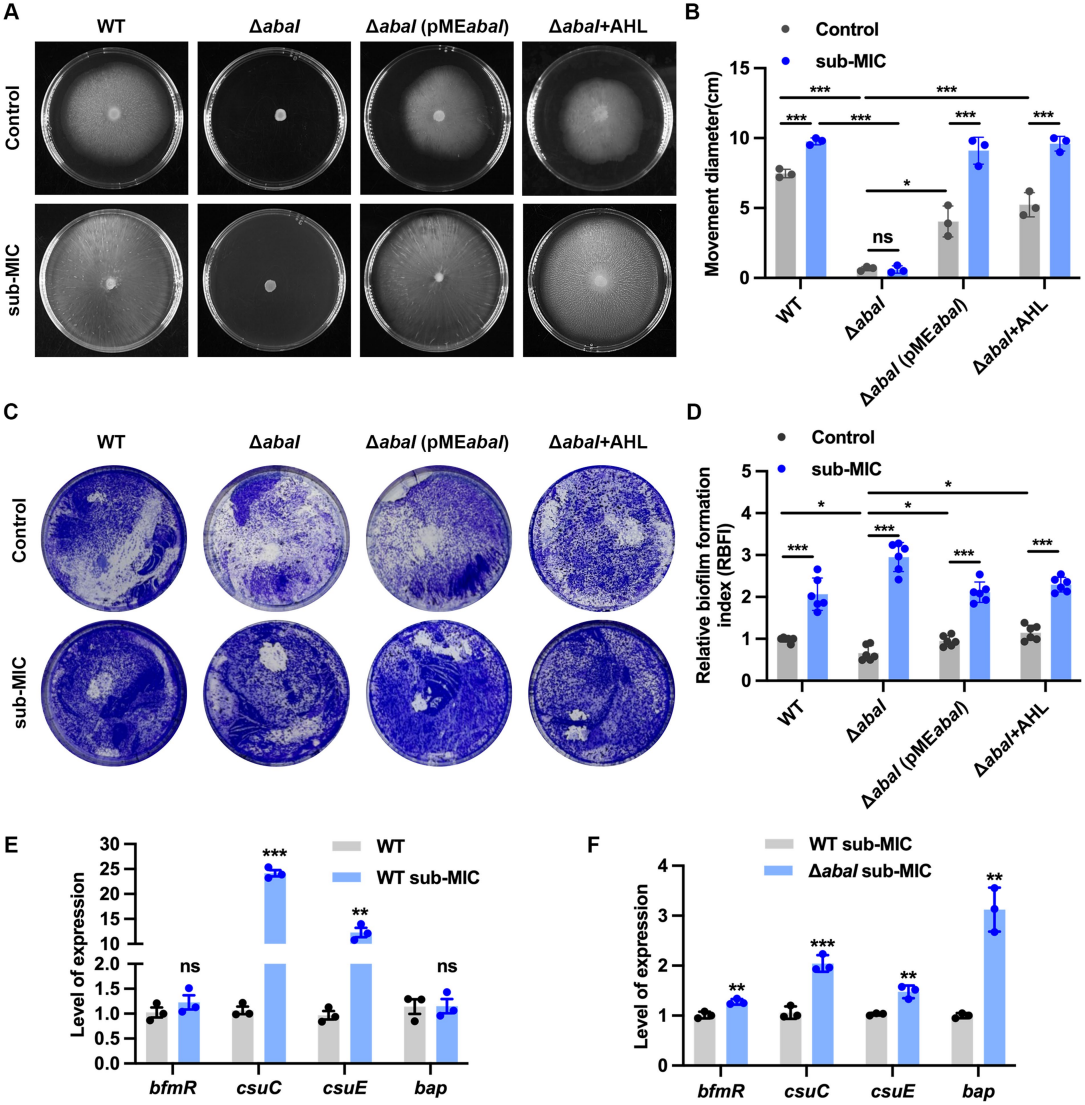
Effects of QS on bacterial surface motility and biofilm formation under antibiotic pressure. **(A)** The distance traveled by the bacteria on the motile plate; **(B)** Diameter of bacterial movement area; **(C)** Bacterial biofilm crystal violet staining; **(D)** Biofilm formation index; **(E)** Expression of genes involved in biofilm formation in WT after sub-MIC meropenem; **(F)** Expression of genes involved in biofilm formation in WT and *ΔabaI* after sub-MIC meropenem. The data are presented as the mean ± SEM. *p < 0.05, **p < 0.01, ***p < 0.001, ns: non-significant.

The surface motility of *A. baumannii* was assessed by measuring the expansion diameter on soft agar plates. Under baseline conditions, the Δ*abaI* strain exhibited markedly impaired motility compared to the wild-type (WT). Following antibiotic treatment, the WT strain showed a significant increase in motility, indicating that sub-inhibitory concentrations of meropenem enhance surface-associated motility. In contrast, antibiotic exposure did not significantly alter motility in the Δ*abaI* mutant. Notably, the Δ*abaI*(pME*abaI*) strain partially restored motility in the Δ*abaI* strain, and showed similar levels to WT, both in the presence and absence of antibiotic pressure. Furthermore, subsequent addition of acyl-homoserine lactone (AHL) also substantially restored motility in the Δ*abaI* strain (Figures 2A,B). These results demonstrate that *abaI* complementation and AHL supplementation can rescue the motility deficit resulting from *abaI* deletion, confirming that QS plays an essential role in regulating surface motility in *A. baumannii* under antibiotic pressure.

### 3.3 Effects of QS on biofilm formation and associated gene expression under antibiotic pressure

We next examined biofilm formation under antibiotic stress. Consistent with the motility results, antibiotic treatment significantly enhanced biofilm production in the WT strain. The Δ*abaI* mutant exhibited reduced biofilm formation under control conditions, further supporting the involvement of QS in this process, the Δ*abaI*(pME*abaI*) complemented strain behaved similarly to the WT strain, restoring biofilm levels compared to the Δ*abaI* strain. Interestingly, while antibiotic exposure enhanced biofilm formation in the WT, it induced an even more pronounced biofilm increase in the Δ*abaI* strain. Subsequent, the Δ*abaI*(pME*abaI*) complemented strain and the AHL supplementation not only reversed this enhancement but reduced biofilm formation to below-baseline levels (Figures 2C,D).

To elucidate the mechanistic basis for these observations, we analyzed the expression of biofilm-associated genes (*csuC, csuE, bap*, and *bfmR*). In the WT strain, antibiotic stress specifically up-regulated *csuC* and *csuE*, while *bap* and *bfmR* expression remained unchanged (Figure 2E). In contrast, the *ΔabaI* strain showed significant up-regulation of all four genes under antibiotic stress (Figure 2F). This distinct gene expression pattern suggests that in the absence of functional QS, antibiotic stress may trigger a compensatory overexpression of biofilm-related genes through alternative regulatory pathways. The precise mechanisms governing these regulatory interactions require further investigation.

### 3.4 Effect of QS on host cell adhesion and invasion under antibiotic pressure

**Figure 3:**
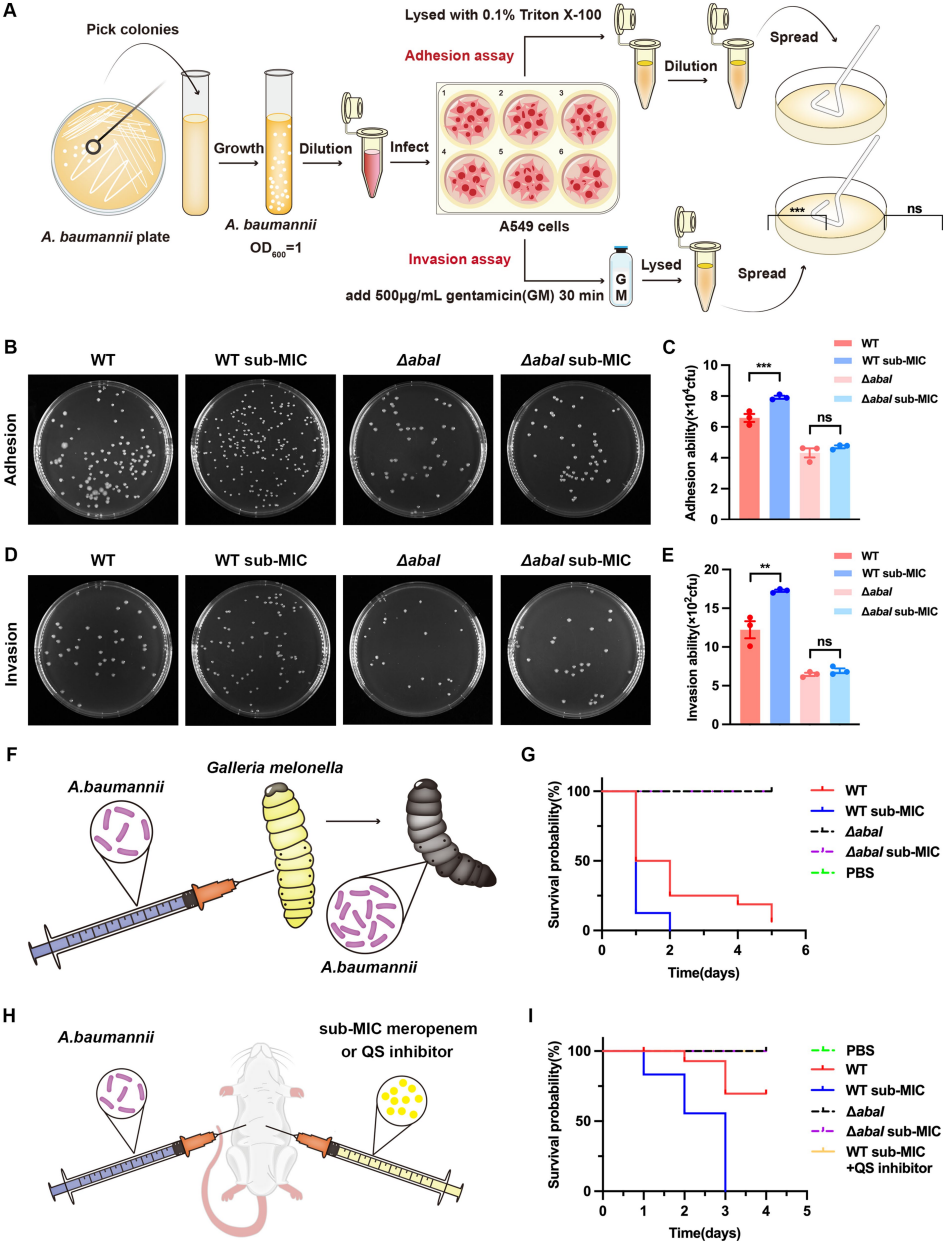
QS regulated virulence of *A. baumannii* under antibiotic pressure. **(A)** Flow chart of *A. baumannii* adhesion and invasion assay on A549 cells; **(B)** the adhesion of bacteria to cells; **(C)** number of bacterial colonies in adhesion assay; **(D)** the invasion of bacteria to cells; **(E)** number of bacterial colonies in the invasion assay; **(F)** flow chart of *A. baumannii* infection of *Galleria mellonella*; **(G)** effect of QS on *Galleria mellonella* virulence under antibiotic pressure. **(H)** Flow chart of *A. baumannii* infection of *mice*; **(I)** effect of QS on *mice* virulence under antibiotic pressure (*n* = 6). The data are presented as the mean ± SEM. **p < 0.01, ***p < 0.001, ns: non-significant.

To examine the role of QS in host cell adhesion and invasion, we infected A549 cells with either WT or Δ*abaI* strain (Figure 3A). The results indicated that the trends in bacterial adhesion and invasion capabilities were generally consistent. Following antibiotic treatment, both adhesion and invasion capacities of the WT strain were significantly enhanced, indicating that antibiotic stress promotes the virulence of *A. baumannii* toward host cells. In contrast, the adhesion and invasion capabilities of the *ΔabaI* strain remained unaltered after antibiotic challenge (Figures 3B–E). These results suggest that *A. baumannii* may employs the QS system to augment its host cell adhesion and invasion under antibiotic pressure.

### 3.5 Effect of QS on virulence in the *galleria mellonella* and mouse infection models under antibiotic pressure

We further assessed virulence using a *Galleria mellonella* infection model. Untreated larvae typically exhibit a pale-yellow coloration, which progressively darkens to black following infection until death occurs (Figure 3F). In a five-day infection assay, larvae inoculated with WT strain showed survival rates of 50% after 24 h, 25% after 48 h, and only 6.25% by day 5. When larvae were infected with antibiotic-treated WT bacteria, all larvae succumbed within 2 days, demonstrating that antibiotic pressure enhances bacterial pathogenicity. In contrast, the Δ*abaI* strain exhibited markedly attenuated virulence, with nearly abrogated lethality (Figure 3G). This reduced virulence was not significantly affected by antibiotic treatment. Together, these findings underscore that QS is essential for the full virulence of *A. baumannii*, and its absence drastically impairs bacterial pathogenicity *in vivo*.

To further validate these findings in a mammalian host and directly address the therapeutic potential of targeting QS, we established a mouse systemic infection model under antibiotic pressure (Figure 3H). Mice were infected with strains pre-exposed to sub-MIC meropenem to simulate a drug-pressure environment. Infection with the antibiotic-pretreated WT strain resulted in 100% mortality within 3 days, a significantly more severe outcome compared to infection with the untreated WT strain (66% survival at 72 h), confirming that antibiotic pressure markedly enhances WT pathogenicity in mammals. In contrast, both the untreated and antibiotic-pretreated Δ*abaI* mutant exhibited virtually no lethality. Critically, to test whether QS inhibition could confer a survival benefit even when antibiotic efficacy is low, a separate group of mice infected with the WT strain was administered a combination of sub-MIC meropenem and the QS inhibitor indomethacin (40 μg/mL). This combination therapy resulted in a significant survival advantage compared to mice receiving sub-MIC meropenem alone (Figure 3I). This finding strongly suggests that pharmacological inhibition of QS can effectively reduce virulence and improve outcomes in a mammalian host, even under conditions where conventional antibiotic monotherapy is failing.

### 3.6 Transcriptome analysis of *Acinetobacter baumannii* under antibiotic pressure

**Figure 4:**
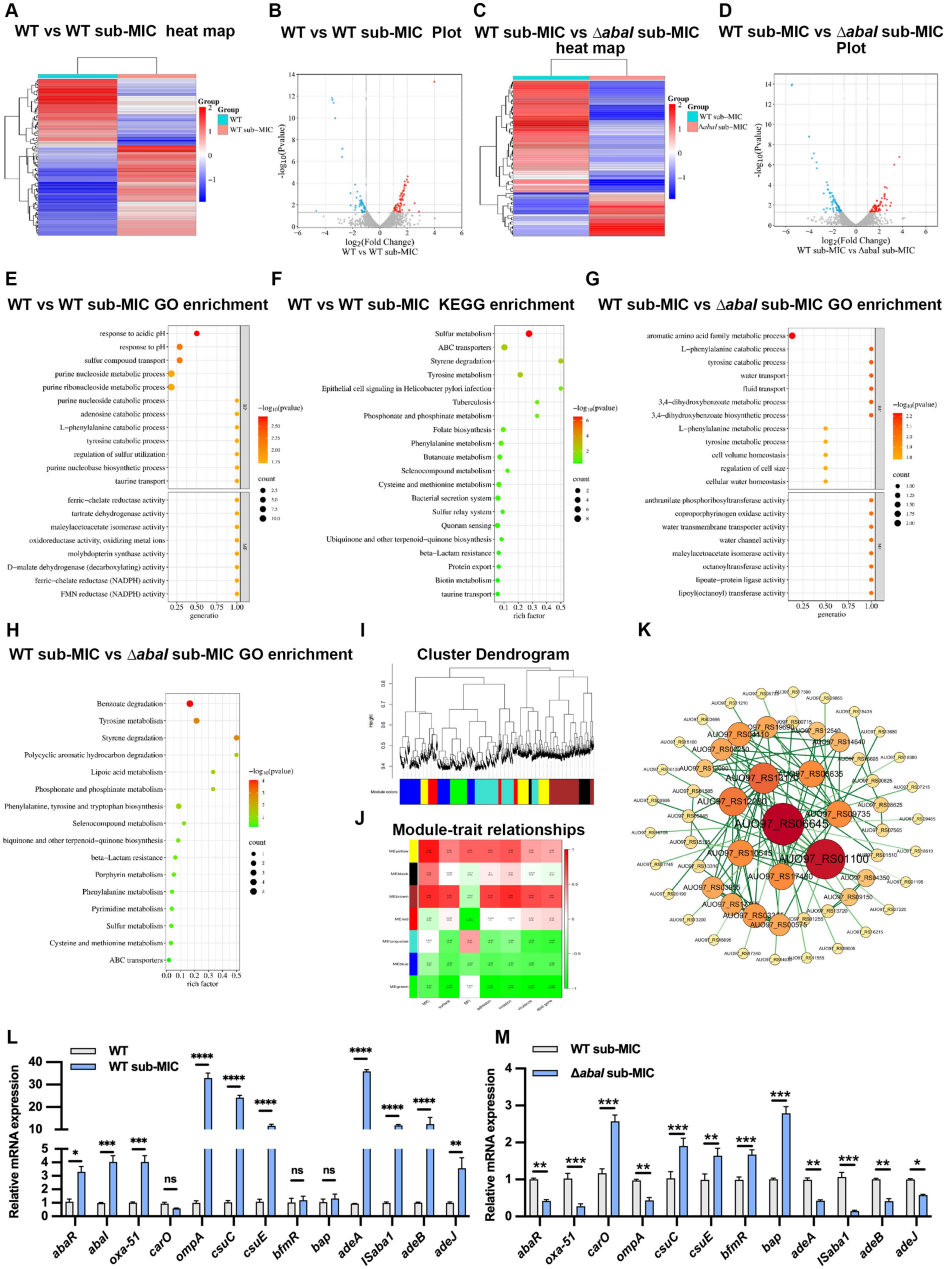
Transcriptome sequencing analysis of changes in *A. baumannii* under antibiotic pressure**. (A)** Heat maps of WT differentially expressed genes before and after sub-MIC meropenem treatment; **(B)** volcano map of WT differentially expressed gene before and after sub-MIC meropenem treatment; **(C)** heat maps of differentially expressed genes between WT and *ΔabaI* after sub-MIC meropenem; **(D)** volcano map of differentially expressed genes between WT and *ΔabaI* after sub-MIC meropenem; **(E)** GO function analysis of WT differentially expressed genes before and after sub-MIC meropenem treatment; **(F)** KEGG enrichment analysis of WT differentially expressed genes before and after sub-MIC meropenem treatment; **(G)** GO function analysis of differentially expressed genes between WT and Δ*abaI* after sub-MIC meropenem; **(H)** KEGG enrichment analysis of differentially expressed genes between WT and Δ*abaI* after sub-MIC meropenem; **(I)** clustering dendrogram of genes, with dissimilarity based on topological overlap, together with assigned module colors; **(J)** module-trait associations; **(K)** the co-expresses network of green module; (L, M) Expression of resistance and virulence-related genes in WT **(L)** and *ΔabaI* **(M)** strains after sub-inhibitory concentration of meropenem treatment. The data are presented as the mean ± SEM. *p < 0.05, **p < 0.01, ***p < 0.001, ****p < 0.001, ns: non-significant.

To elucidate the regulatory role of the QS system in bacterial adaptation to antibiotic stress, we performed transcriptomic sequencing of WT and *ΔabaI* strains with or without sub-MIC antibiotic treatment. Differentially expressed genes (DEGs) were identified using thresholds of |log_2_FoldChange| > 1, and *p*-value < 0.05. Compared to the untreated WT group, the WT sub-MIC group exhibited significant expression changes in 111 genes, with 64 up-regulated and 47 down-regulated (Figures 4A,B). Between the WT sub-MIC and Δ*abaI* sub-MIC groups, 71 genes were differentially expressed, including 20 up-regulated and 51 down-regulated (Figures 4C,D).

Gene Ontology (GO) and KEGG pathway enrichment analyses of DEGs between WT and WT sub-MIC groups revealed significant enrichment in biological processes including response to pH and sulfur compound transport, as well as pathways including sulfur metabolism, ABC transporters, and quorum sensing (Figures 4E,F), underscoring the potential involvement of QS in antibiotic stress adaptation. In contrast, DEGs between WT and *ΔabaI* strains under antibiotic treatment were mainly enriched in GO terms such as L-phenylalanine catabolic process, water transport, and KEGG pathways such as beta-lactam resistance, tyrosine metabolism, and benzoate degradation (Figures 4G,H), suggesting that QS may modulate antibiotic resistance and virulence through these mechanisms.

We further performed weighted gene co-expression network analysis (WGCNA) to identify gene modules associated with bacterial phenotypes. A total of 3,680 genes were clustered into seven distinct modules based on expression patterns (Figure 4I). Correlation analysis between module eigengenes and measured phenotypes revealed that the green module showed the strongest association with motility, adhesion, virulence, and quorum sensing genes (Figure 4J). Further refinement of the green module network using Cytoscape identified 58 nodes and 99 edges after topological filtering. Among these, the most highly connected hub genes included AUO97_RS06645 (*abaI*), AUO97_RS01100 (*bap*), along with some other central regulators (Figure 4K), indicating their crucial roles in the adaptive response network.

To validate the transcriptomic data obtained from RNA sequencing, we performed qRT-PCR analysis on selected genes known to be associated with bacterial resistance and virulence. The results showed that, compared to the WT group, the expression levels of *abaR, abaI, oxa-51, adeA, adeB, adeJ, IsAba1, ompA, csuC*, and *csuE* genes were significantly up-regulated in the WT sub-MIC group, whereas the expression of *carO, bap* and *bfmR* genes did not change significantly (Figure 4L). In contrast, when comparing the *ΔabaI* sub-MIC strain to the WT sub-MIC group, the expression levels of *abaR, oxa-51, ompA, adeA, adeB, adeJ*, and *IsAba1* genes were significantly down-regulated, while *carO, csuC, csuE, bap*, and *bfmR* genes were markedly up-regulated (Figure 4M). These gene expression patterns align with the phenotypic observations described earlier in this study: the enhanced resistance and virulence of the WT sub-MIC group relative to the WT group, as well as the phenotypic divergence between the *ΔabaI* and WT strains under antibiotic pressure. The expression trends detected by qRT-PCR were consistent with the RNA-seq results, confirming the reliability of the transcriptomic data. Minor discrepancies in expression values may be attributed to factors such as RNA degradation, batch effects across samples, or limited sample size.

## 4 Discussion

### 4.1 Antibiotic pressure and adaptive challenges in *Acinetobacter baumannii*

*Acinetobacter baumannii* has emerged as a major nosocomial pathogen, capable of persisting in both hospital environments and natural reservoirs, thereby posing a substantial threat to public health. Antibiotic therapy remains a primary intervention against *A. baumannii* infections; however, the widespread use of antibiotics has led to their accumulation in various ecological niches, frequently resulting in bacterial exposure to sub-inhibitory concentrations of antimicrobial drugs. Substantial evidence indicates that sub-MIC antibiotics can profoundly affect bacterial behavior, including virulence expression and resistance evolution, thereby exacerbating the challenge of antimicrobial resistance. In our study, antibiotic pressure at sub-MIC enhanced the virulence of WT strain of *A. baumannii*, and this adaptive response was strictly dependent on its QS system. The Δ*abaI* mutant strain was not only attenuated in basal virulence, but also insensitive to antibiotic pressure. Despite this, the adaptive mechanisms underlying resistance and virulence evolution in *A. baumannii* under antibiotic pressure remain poorly elucidated.

### 4.2 Quorum sensing as a therapeutic target

Bacteria employ quorum sensing (QS) to coordinate critical physiological processes and adapt to environmental stresses (Saipriya et al., 2020). Targeting QS mechanisms represents a promising strategy for mitigating virulence and resistance in multidrug-resistant pathogens, potentially offering novel therapeutic avenues. Our previous work, utilizing cellular and animal models including mice, *Galleria mellonella*, and zebrafish, established the involvement of the AbaI/AHL QS system in the infectivity and pathogenicity of *A. baumannii* (Sun et al., 2021; Jiang et al., 2024). Moreover, previous studies have shown the multifaceted role of *abaI* genes in the regulation of *A. baumannii*, highlighting the central role of *abaI* in its physiology, pathogenesis and antibiotic resistance (Pumirat et al., 2024). Building upon these findings, the current study aimed to elucidate the role of the AbaI/AHL system in the adaptive responses of *A. baumannii* to antibiotic stress.

### 4.3 QS modulates antimicrobial resistance and adaptive evolution

We initially investigated the influence of QS on antimicrobial resistance in *A. baumannii*. In this study, the Δ*abaI* mutant exhibited significantly reduced resistance to multiple antibiotics-including penicillin, streptomycin, gentamicin, tetracycline, ciprofloxacin, ceftazidime, piperacillin-tazobactam and meropenem, compared to the wild-type strain. This observed link between a functional QS system and enhanced antimicrobial resistance is further supported by evidence from clinical strains (Tang et al., 2020). Building upon this finding, we further investigated whether QS contributes to the adaptive evolution of resistance under antibiotic pressure—a phenomenon widely documented in various bacterial species (Gutierrez et al., 2013; Fernandes et al., 2025). Consistent with this paradigm, our data demonstrate that the AbaI-dependent QS system of *A. baumannii* acts as a key driver of rapid adaptive evolution. The *ΔabaI* mutant failed to elevate its MIC under sustained antibiotic pressure—while the WT strain did so dramatically—suggests that QS is not merely a virulence regulator but may also be essential for resistance evolution. This divergence clearly indicates that the QS system is indispensable for the rapid optimization of resistance under antibiotic selection pressure. We propose that QS enables a coordinated population-level response, potentially facilitating the regulation of resistance genes (e.g., those encoding efflux pumps or antibiotic-inactivating enzymes) in a cell-density-dependent manner (Fernandes et al., 2025; Lee et al., 2017). Such coordination may accelerate the selection and fixation of beneficial mutations, enhancing the evolvability of *A. baumannii* in challenging environments.

QS system is not merely a virulence coordinator; it functions as a central hub that integrates antibiotic stress signals and rapidly translates them into adaptive resistance. Recent evidence indicates that the LasRI, RhlRI, and PQS circuits of *Pseudomonas aeruginosa* can sense sub-lethal concentrations of β-lactams or aminoglycosides and, in response, up-regulate efflux pumps, reduce porin expression and increase mutagenic DNA repair, thereby accelerating the fixation of resistance mutations (Guo et al., 2024). Conversely, the host has evolved glyco-based counter-measures: salivary MUC5B O-glycans suppress the quorum-sensing pathways of *Streptococcus mutans*, blocking the development of natural competence and consequently preventing the acquisition of exogenous resistance determinants via transformation (Werlang et al., 2021). This non-bactericidal, glycan-mediated interference with QS circuitry highlights a potential host strategy to limit bacterial adaptability without exerting selective pressure for resistance—a concept that may inspire future exploration of whether similar glyco-based interventions could modulate QS-driven resistance evolution in *A. baumannii*. Collectively, these findings position quorum sensing as a core mediator of bacterial antibiotic adaptation and validate QS-network interference as a promising route to subvert the evolutionary trajectory of antimicrobial resistance.

### 4.4 Quorum sensing modulates motility and biofilm formation under antibiotic stress

Surface-associated motility is closely related to bacterial adhesion, colonization, and biofilm formation (Ye et al., 2024), and its impairment significantly attenuates *A. baumannii* pathogenicity (Ko et al., 2023; Armalyte et al., 2023)_._ In line with previous findings (Lin et al., 2023), we observed that the Δ*abaI* mutant exhibited markedly impaired motility under baseline conditions. Notably, we found that sub-MIC meropenem exposure selectively enhanced motility in the WT strain, whereas this adaptive response was completely absent in the Δ*abaI* mutant. However, motility was substantially restored upon exogenous gene complemented and AHL supplementation, confirming that AHL-mediated QS is essential for regulating surface motility under antibiotic stress. These results suggest that QS may facilitate surface spreading in response to antibiotic challenge, possibly promoting escape from localized stress and colonization of new niches (Su et al., 2023).

Biofilm formation is a key determinant of *A. baumannii*’s persistence in clinical settings (Cavallo et al., 2023). Consistent with earlier reports (Seleem et al., 2020), disruption of quorum sensing via *abaI* deletion reduced biofilm formation under standard conditions. Interestingly, sub-MIC meropenem enhanced biofilm production in both WT and Δ*abaI* strains, with the mutant exhibiting a more pronounced increase—a hyper-biofilm phenotype that was reversed upon AHL complementation. This suggests that in the absence of QS, antibiotic stress may trigger compensatory mechanisms enhancing biofilm production, possibly as a survival strategy. The reversal upon AHL addition confirms the specificity of the response and this interpretation is directly supported by our transcriptome data to underscores the role of QS in fine-tuning this process.

To elucidate the mechanistic basis for the observed biofilm phenotypes, we analyzed the expression of key biofilm-associated genes. In wild-type cells, sub-MIC meropenem specifically induced the upregulation of *csuC* and *csuE*, genes encoding structural components of type I pili that are crucial for surface attachment and early biofilm development. This finding is consistent with their established role in initial biofilm formation (Kim et al., 2022). In striking contrast, the Δ*abaI* mutant exhibited broad transcriptional dysregulation, with significant upregulation of all four genes tested (*csuC*, *csuE*, *bap*, and *bfmR*). Given that BfmR is a known positive regulator of the *csu* operon and Bap is a key protein for biofilm maturation (Kim et al., 2022), this concerted overexpression suggests that the absence of QS triggers a compensatory response, potentially via the activation of alternative regulatory systems such as BfmRS, to promote biofilm formation under antibiotic stress.

This critical role of QS as a master regulator of biofilm architecture is not unique to *A. baumannii* but reflects an evolutionarily conserved strategy among Gram-negative pathogens. For instance, in *Pseudomonas aeruginosa*, inhibition of its PQS or AHL-based QS systems—whether by specific compounds like wogonin or natural plant extracts—robustly disrupts biofilm integrity and inhibits motility (Tang et al., 2024; Wang et al., 2021; Rather et al., 2021). The broad upregulation of biofilm genes in our QS-deficient mutant aligns with observations in other bacterial systems, where the loss of a primary regulatory hub like QS can lead to dysregulated activation of compensatory pathways. In *Serratia marcescens*, for example, the absence of canonical QS leads to altered expression of genes controlled by alternative global regulators, such as RsmA (Quintero-Yanes et al., 2019). Similarly, we propose that in *A. baumannii*, the AbaI/R QS system normally acts as a transcriptional modulator that fine-tunes the expression of biofilm-related genes. Its absence disrupts this homeostatic control, leading to aberrant activation of stress-responsive regulators.

Our transcriptomic and phenotypic data indicate that in the absence of the canonical AbaI/R QS system, antibiotic stress triggers a pronounced hyper-biofilm response accompanied by broad upregulation of biofilm-related genes (including *bfmR* and *bap*). This pattern strongly suggests the activation of compensatory regulatory networks, with the BfmRS two-component system being a prime candidate. Having established the central adaptive role of QS, the precise hierarchy and crosstalk between the QS system and these alternative pathways emerge as a key question for future investigation. Future work employing genetic tools such as Δ*abaI*Δ*bfmR* double mutants will be essential to directly test this compensatory model and to unravel the systems-level redundancy that enables *A. baumannii* to maintain adaptive plasticity under selective pressure.

### 4.5 QS modulates host-pathogen interactions under antibiotic pressure

Adhesion to and invasion of host epithelial cells are critical early steps in *A. baumannii* pathogenesis (Choi et al., 2008)_._ Our results demonstrate that sub-MIC meropenem enhanced both adhesion and invasion in the WT strain, but not in the Δ*abaI* mutant. These findings imply that the AbaI/AHL system may potentiate virulence traits under antibiotic pressure, possibly through upregulation of adhesins or modulation of surface properties. It is important to note that this enhanced adhesion invasion, mediated by QS, observed *in vitro*, closely matches the lethal phenotype observed in our *in vivo* infection models. This highlights the role of QS as a bridge between antibiotic stress and host infection efficiency.

The *Galleria mellonella* model has been widely used to study bacterial pathogenesis (Tsai et al., 2016; Menard et al., 2021). Our results showed that the Δ*abaI* mutant was virtually avirulent, underscoring the essential role of QS in virulence. Sub-MIC meropenem significantly enhanced the lethality of the WT strain but did not alter the avirulent phenotype of the mutant. The strong positive correlation between *Galleria mellonella* killing and bacterial pathogenicity traits supports the conclusion that QS is a central regulator of antibiotic-induced virulence in *A. baumannii*.

The mouse systemic infection model provided critical evidence for our assessment of the pathogenic role of the QS system in a mammalian host. Consistent with the observations in the *Galleria mellonella* model, the Δ*abaI* mutant showed significantly attenuated virulence in mice, further confirming that QS is essential for systemic infection by *A. baumannii*. Notably, pretreatment with a sub-inhibitory concentration of meropenem markedly accelerated mortality in mice infected with the WT strain, yet failed to restore virulence in the Δ*abaI* mutant. These *in vivo* findings underscore that QS disruption effectively abolishes pathogenicity even under antibiotic pressure, highlighting its potential as a therapeutic target in infections where conventional antibiotics may inadvertently exacerbate virulence.

### 4.6 Systems-level insights into QS-mediated adaptation

Transcriptomic analysis revealed that sub-MIC meropenem exposure induced differential expression in the WT strain, with significant enrichment in pathways involved in pH response, sulfur metabolism, and quorum sensing. In contrast, comparative analysis between antibiotic-treated WT and Δ*abaI* strains highlighted enrichment in ABC transporters, β-lactam resistance, and aromatic amino acid biosynthesis—pathways critically associated with antibiotic resistance and host adaptation. These findings suggest that QS modulates bacterial adaptation to antibiotic stress through pleiotropic regulation of metabolic and resistance pathways, fine-tuning resource allocation among survival, defense, and virulence mechanisms. The influence of QS extends to metabolic reprogramming processes such as sulfur assimilation and ABC transporter activity, further underscoring its central role in promoting bacterial survival under adverse environmental conditions. Weighted gene co-expression network analysis (WGCNA) clustered transcripts into seven modules, one of which—the green module—showed the strongest correlation with key phenotypes including MIC, motility, biofilm formation, adhesion, invasion, and virulence. Notably, hub genes within this module included *abaI*, *abaR*, and *bap*, placing QS components and biofilm-associated factors at the core of the adaptive regulatory network. This systems-level insight confirms that QS functions as a master regulator orchestrating diverse adaptive responses to antibiotic stress, and highlights its potential as a multi-target therapeutic avenue.

## 5 Conclusion

In conclusion, our integrative analysis demonstrates that the AbaI/AHL QS system lies at the heart of *A. baumannii*’s adaptive response to antibiotic stress, governing resistance evolution, virulence expression, and metabolic remodeling. Disrupting QS signaling abrogates these adaptive responses, underscoring its therapeutic potential. Future efforts aimed at developing anti-virulence strategies targeting the QS system may offer novel means to curb resistance evolution and mitigate infections caused by this multidrug-resistant pathogen.

## Data Availability

The genome sequencing data from this study have been deposited in the NCBI database, and the accession number is PRJNA1344818.
