# Supplementary material for: Regulation of drug resistance and virulence of Acinetobacter baumannii by quorum sensing system under antibiotic pressure
Source: Front Microbiol. 2026 Jan 29;17:1744356. doi: 10.3389/fmicb.2026.1744356 (PMC12894222; doi:10.3389/fmicb.2026.1744356)
Supplement: Supplementary file 1 [file Table_1.docx]

**Supplementary Material**

**Regulation of drug resistance and virulence of *Acinetobacter baumannii* by Quorum Sensing system under antibiotic pressure**

**Xingyu Jiang^1#^, Xuchun Shan^1#^, Xiaomeng Yang^1^, Xin Zhang^1^, Yang Xiang^2^, Yan Chen^3*^, Zhaohui Ni^1*^**

1 Department of Pathogen Biology, The Key Laboratory of Zoonosis, Chinese Ministry of Education, College of Basic Medical Sciences, Jilin University, Changchun, China;

2 The First Hospital of Jilin University, Changchun, China;

3 Department of Neurosurgery, The Second Hospital of Jilin University, Changchun, China.

#Contributed equally.

*** Correspondence:**

**Zhaohui Ni: nich@jlu.edu.cn; Yan Chen: drchenyan@jlu.edu.cn**

**Table S1. Bacterial strains used in this study.**

| Strain | Relevant characteristics | Reference or source |
| --- | --- | --- |
| ATCC 17978 (WT) | *Acinetobacter baumannii* standard strain | Ayush Kumar |
| ΔabaI | WT with deletion in abaI gene | Our laboratory |
| ΔabaI(pMEabaI) | ΔabaI harboring pME6032, containing the abaI gene | Our laboratory |

**Table S2. Primers used in this study**

| Gene | Primer sequence (5’- 3’) |
| --- | --- |
| 16S Forward | CAGCTCGTGTCGTGAGATGT |
| 16S Reverse | CGTAAGGGCCATGATGACTT |
| *abaR* Forward | CGCATCAAGGCTCGGATTTG |
| *abaR* Reverse | CGTTTCTGCCATGCTTCTGG |
| *abaI* Forward | CAGGCTGTGTCATCACCTGT |
| *abaI* Reverse | TTTGCGCCTTGTTCTCTTGC |
| *bap* Forward | GGTAACCTGGTGGATGGCGATAC |
| *bap* Reverse | GTGCTGTGATGTCTGCTGAAACTG |
| *csuC* Forward | ATTATACCAAGCCCGCCTGTAGC |
| *csuC* Reverse | GACGATATGACTGCTCTTTGCCATC |
| *csuE* Forward | TGGGAATCGGTGCCTTCTTTGAG |
| *csuE* Reverse | TGAGAGTGAACCAAGCGAGTCTG |
| *BfmR* Forward | CGCCCACACTATCATCAACCAATC |
| *BfmR* Reverse | CATGACCACCCTTGCCTTC |
| *adeJ* Forward | GTACGCCACCAAGTACACCA |
| *adeJ* Reverse | GACATAATCGTCTGCACCCATTTCC |
| *adeA* Forward | CTGAAGCTGAGGTGGCAAGA |
| *adeA* Reverse | TTTTGTCTGGCCAGCAATGC |
| *adeB* Forward | ACGTCATCGGGTGAAGCAAT |
| *adeB* Reverse | TACCTGTCCACTCGTAGCCA |

**Table S3. Minimum inhibitory concentrations of antibiotics used in this study.**

| Antibiotic | MIC(µg/mL) | | |
| --- | --- | --- | --- |
|  | WT | ∆*abaI* | ΔabaI(pMEabaI) |
| Gentamicin | 4 | 1 | 2 |
| Penicillin | 128 | 64 | 64 |
| Streptomycin | 32 | 16 | 32 |
| Meropenem | 1 | 0.5 | 1 |
| Ciprofloxacin | 2 | 1 | 2 |
| Ceftazidime | 8 | 2 | 4 |
| Piperacillin-tazobactam | 8 | 8 | 8 |
| Tetracycline | 2 | 2 | 2 |
